# Cooling spray or lidocaine spray and needle insertion pain in hemodialysis patients: an open-label cross-over randomized clinical trial

**DOI:** 10.1186/s12871-023-02028-w

**Published:** 2023-03-07

**Authors:** Armin Khosravi Pour, Sima Hejazi, Ahmad Kameli, Tooba Hoseini Azizi, Mohammad Reza Armat, Maesoomeh Eshghi

**Affiliations:** 1 Student Research Committee, Bojnurd Faculty of Nursing, North Khorasan University of Medical Sciences, Bojnurd, Iran; 2 Department of Nursing, Bojnurd Faculty of Nursing, North Khorasan University of Medical Sciences, Shahriar Street, Bojnurd, Iran; 3 Department of Disaster Public Health, School of Public Health, Tehran University of Medical Sciences, Tehran, Iran; 4 Student Research Committee, Bojnurd Faculty of Nursing, North Khorasan University of Medical Sciences, Bojnurd, Iran

**Keywords:** Pain, Hemodialysis, Vapocoolant Spray, Lidocaine, Cross over Design

## Abstract

**Background:**

The needle insertion pain to perform hemodialysis is the main challenge and a common problem that requires pain management techniques for patients’ comfort.

**Aim:**

This study aimed to compare the effects of cooling and lidocaine sprays on needle insertion pain in hemodialysis patients.

**Methods:**

In this randomized cross-over clinical trial study, the hemodialysis patients were selected through convenience sampling according to inclusion criteria and randomly assigned to three intervention groups using the block randomization method. Each patient received three interventions in a cross-over design: Cooling spray or 10% lidocaine spray or placebo spray. There was a 2-week wash-out time between each intervention. The pain score was measured four times for each patient by the Numerical Rating Scale.

**Results:**

Forty-one hemodialysis patients were included. The results showed a significant interaction between time and group (p < 0.05), so only observations of time 1 with adjustment for baseline values were used to evaluate the effect of the intervention. Patients receiving cooling spray reported 2.29 less pain score on average compared to placebo (B=-2.29, 95% CI: -4.17 to -0.43; p < 0.05); Also, patients receiving cooling spray reported a 1.61 lower pain score than those receiving lidocaine spray, but this difference was not statistically significant (95% CI: -0.26 to 3.48; p > 0.05).

**Conclusion:**

The cooling spray was effective in reducing the needle insertion pain. Although it was impossible to compare the pain scores at different times and following different interventions, the present study results can help supplement the existing knowledge regarding cooling and lidocaine sprays.

## Introduction

Pain is defined as an unpleasant sensation and emotional experience [1], and it can even be considered a disease [2]. Pain can slow the patient’s recovery and increase the family’s fatigue and discomfort [3]. Although intravascular cannulation is a common painful procedure, no analgesic is routinely used [4]. Research shows that 5.3% and 22% of adults have a severe and moderate fear of needles, respectively [5]. Nearly two million patients worldwide are dialysis-dependent, and of these, approximately 90% receive hemodialysis (HD) three times weekly [6].

The HD is the most frequently used renal replacement therapy, and an arteriovenous fistula (AVF) is the gold standard for vascular access in these patients [6]. It is true for HD patients who experience a large-diameter needle insertion in the fistula area two to three times a week and about 300 times a year [7]. Therefore, the pain of the needle insertion to AVFs to perform HD is unbearable and a highly prevalent problem [8, 9] that requires pain management techniques for long-term treatment and patients’ comfort [6]. More than one-fifth of HD patients report this pain as unbearable. Many of these patients consider needle insertion before dialysis the most stressful part of treatment and the most significant concern during dialysis [10]. This pain can reduce the quality of life [11], is a factor for anxiety and stress, and underlies mental illnesses and depression in these patients [12] [13].

On the other hand, a painful procedure can instill a sense of distrust in medical staff; the patient’s pain intolerance also increases the risk of occupational accidents, such as inserting an infected needle in the nurse’s hand [14, 15]. Therefore, pain control in these patients seems necessary and is considered one of the critical tasks of medical and health staff. A nurse in direct contact with HD patients plays an essential role in pain control [10].

For pain reduction, some methods have been suggested and studied, including the use of topical anesthetics, such as lidocaine [16], distraction techniques [3], producing vibration in the tissue of the injection site [17], imposing pressure [18], thermotherapy [19], and cooling the injection site [20, 21]. Some effective pharmaceutical methods for reducing patients’ pain are topical anesthesia techniques, including gels, ointments, patches, and topical anesthetic sprays, which can reduce the pain caused by medical procedures, such as venipuncture [10]. Choosing the best type and form of analgesic drugs depends on the prescription method and the required effect duration [22] [23]. In addition, the medication should have fewer complications, be simple to use, have a reasonable price, and not interfere with care and treatment activities [24].

The injectable anesthetics are not used for venipuncture or intramuscular injection alone because their injection is as painful as venipuncture or injection [25]. Anesthetic gels and ointments can be superior to injectable anesthetics because they do not induce painful stimuli [16, 26]. However, it should be noted that these ointments penetrate healthy skin and provide analgesic properties on the skin surface layers (in several millimeters), which, to be effective, must be applied to the desired site one hour before [23]. It is necessary to mention that these agents can lead to complications, such as allergic symptoms, fever and rash, shortness of breath, skin whitening in the area of use, redness of the prescription site, and edema [23].

Another form of topical analgesic includes sprays. One of the most well-known types of these sprays is lidocaine spray. Lidocaine spray is used for the topical anesthesia of mucous membranes and skin. The beginning of its practical effect and its easy-to-use method has led to its widespread use, compared to other methods [27]. A new type of spray used in pain control studies is cooling spray. Vapocoolant sprays temporarily and rapidly interrupt pain transmission by lowering the skin surface temperature, reducing the sensitivity of pain receptors, and interfering with ion channel valves in pain transmission, causing topical skin anesthesia [16]. Ethyl chloride (i.e., the active ingredient in many cooling sprays) and other cooling agents are new and alternative analgesics used to induce rapid anesthesia during vascular access in emergency conditions [21]. These sprays have advantages over other mentioned methods, including their immediate effect compared to anesthetic ointments, no need for injection to produce an effect, and reduced risk of needle sticking in staff’s hands. In addition to the materials mentioned above, this spray type has more advantages than other methods, such as distraction and anesthetic ointments [28, 29]. However, these sprays’ analgesia effect is still controversial and contradictory [21, 29].

Furthermore, the existing evidence on the usefulness of cooling sprays is of medium quality and cannot be relied on [21]. Therefore, this study aimed to provide higher-quality evidence. There is a limited number of studies comparing lidocaine and cooling sprays in terms of their pain-reducing effect. Therefore, the present study aimed to compare the effect of cooling and lidocaine sprays on pain induced by needle insertion in HD patients.

## Methods

### Study design and setting

This study was a single-center, open-label, randomized, three-period, three-treatment, sequence crossover clinical trial carried out within May 2019 to October 2019. The research setting was the Hemodialysis Ward of Imam Ali hospital in Bojnurd, North Khorasan province, Iran.

### Population and sampling method

The research population included all patients under treatment with HD, meeting inclusion criteria, and hemodialyzed in the Hemodialysis Center of Imam Ali hospital in Bojnurd, Iran, through an AVF. The inclusion criteria included age of over 18 years, alertness, the ability to speak and understand Persian or the presence of a Persian-speaking translator, no pacemaker or known heart problem, the onset of HD at least 3 months ago, no known allergies to lidocaine, no damaged skin, no use of analgesics and opioids during the last 24 h, no history of using lidocaine cream or products in the past, HD through an AVF, no history of antiarrhythmic drugs (especially class Ш, such as amiodarone), and HD three times a week. The exclusion criteria included the occurrence of skin allergy or abnormal reaction at the vascular access site, not referring for HD in the next session, and lack of venipuncture with the first attempt in each stage.

The convenience sampling method was used in this study. With the effect size of 0.81 obtained from Asgari et al.’s [10] study, using G-Power software (version 3.1.2.3), and taking into account the sample loss, the sample size was estimated to be 41 subjects. All patients under treatment with HD in Imam Ali hospital of Bojnurd who met the inclusion criteria were randomly allocated to three intervention groups via the block randomization method in such a way that first, the blocks ABC, BAC, ACB, BCA, CAB, and CBA were considered. Numbers 0 to 5 were assigned to each block, respectively (A: the cooling spray group, B: the lidocaine spray group, and C: the placebo spray group). Then, from the table of random numbers, a series of random numbers was selected as many as the desired sample numbers (in each group). After deleting numbers 6 to 9, each number was assigned to the corresponding block; then, they were numbered, and the patients were assigned to one of the three groups according to the list. The second author generated the random allocation sequence (SH). The enrolment and assignment of the participants were performed by the sixth author (ME).

### Data collection and interventions

**Fig. 1:**
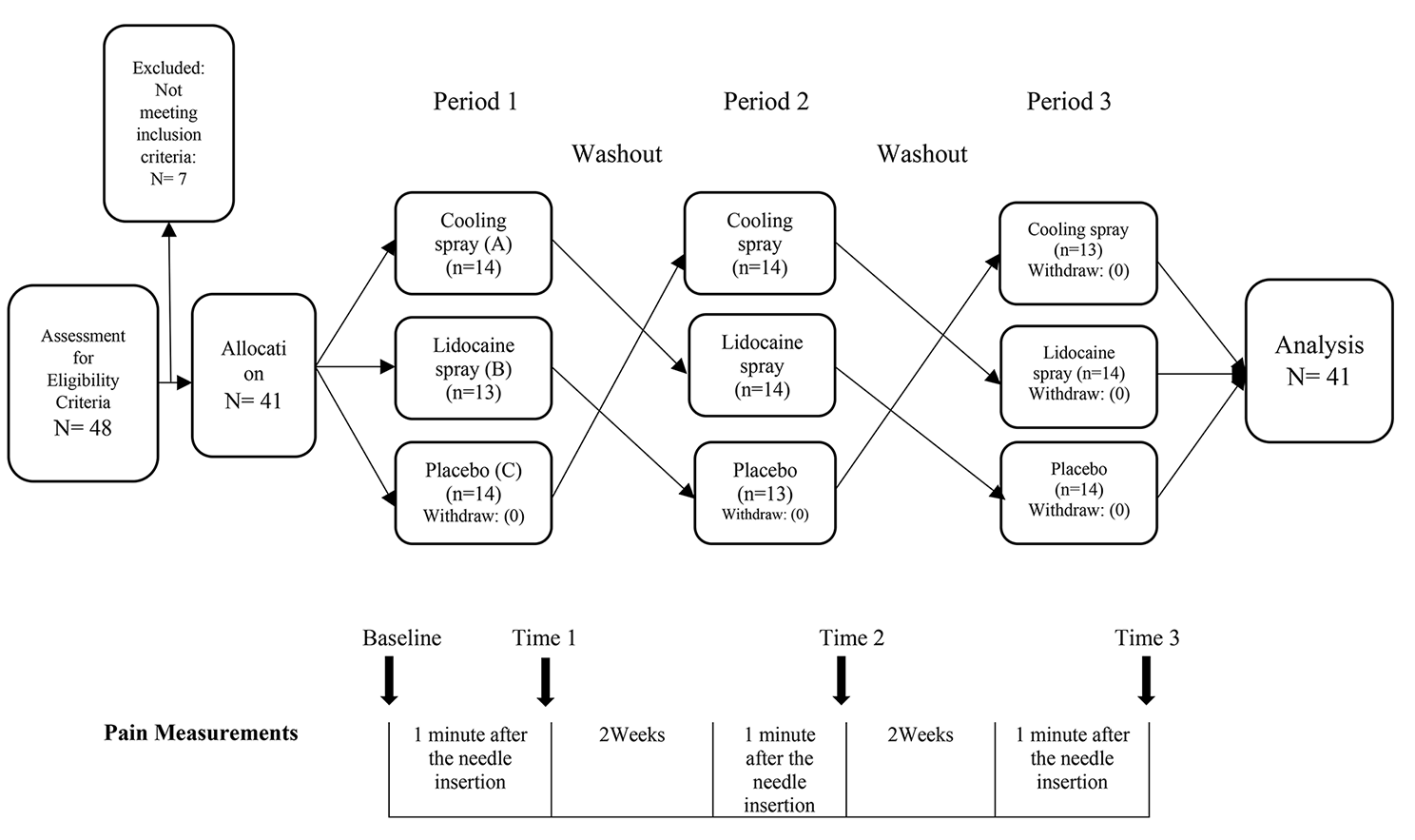
The design of the study

Before the first intervention, informed and written consent was obtained from the patients, and the pain rating scale was trained. The first needle insertion was considered the primary intervention for all patients, and the vascular access procedure was performed without any intervention. The pain score was determined and recorded one minute after needle insertion by the Numerical Rating Scale (NRS). Each patient received one of the interventions in the next HD session (2 days later), including cooling spray (ethyl chloride), 10% lidocaine spray, or placebo spray. Patients who first received a cooling spray (i.e., 2 days after the initial non-intervention needle insertion) received lidocaine spray 2 weeks later and placebo spray 4 weeks later. Patients who first received lidocaine spray received the placebo spray 2 weeks later and the cooling spray 4 weeks later. Patients who first received placebo spray received cooling spray 2 weeks later and lidocaine spray 4 weeks later [29, 30] (Fig. 1).

In the case of using a cooling spray, after disinfecting the skin, the spray was sprayed on the skin for 3 to 4 s at a distance of 15 to 20 cm, allowing the spray to evaporate from the skin for 10 s, and vascular access occurred after the disinfection of skin [31, 32]. In the case of using a lidocaine spray, two puffs of lidocaine spray (20 mg) were sprayed on the skin from a distance of approximately 5 cm. Because topical anesthesia caused by lidocaine occurs within 1 to 5 min after use, 5 min after spraying it on the skin, the skin is disinfected, and the vascular access procedure is performed by the ward nurse [10]. In the case of using a placebo spray, two puffs were sprayed from a distance of 5 cm on the skin, and 5 min after spraying it on the skin and after disinfecting the site, the vascular access procedure was performed [10].

A particular container for lidocaine spray was emptied, washed, and filled with drinking water to prepare the placebo spray. All three sprays’ exterior parts were covered with paper to look almost identical. In all three cases, 1 min after vascular access, the patient’s pain score was determined by asking the patient and using the NRS [10]. In all cases, the design researcher (ME) applied the spray to the patient’s skin, and the design executor (AKh) asked for the pain score without being informed of the spray type. The same arteriovenous needle with size 16 was used to establish the venous route in all patients. In four periods of pain score evaluation in each patient, a specific and identical nurse performed a venipuncture. The nurse and the patient were not informed of the type of spray used. In both lidocaine and cooling sprays, the injection site was examined before and 2 h after each intervention for redness, paleness, edema, cold-induced damage, thrombophlebitis, and skin reactions. Additionally, the patients were trained about the symptoms to report them immediately. No complications occurred for patients.

### Outcome measurement

The research tools included a demographic and disease information questionnaire and the NRS. An identical watch was used to measure time at all stages. The demographic and disease information questionnaire, including age, gender, addiction status, cause of illness, duration of HD onset, and comorbidities, was completed through interviews and the research unit file. The NRS measured the study’s primary outcome, pain during needle insertion. This scale is a valid, short, and easy tool for the assessment of pain, rated within 0–10. A score of 0 is the characteristic scale of lack of pain, and a score of 10 is the characteristic of the most severe pain felt by the patient. The validity and reliability of the NRS have been investigated and proven in several studies [10, 21].

### Data analysis

This study utilized a crossover design trial; accordingly, for each person, the related-to-each other and longitudinal data were recorded; therefore, a linear mixed-effect (LME) model was used to analyze the data and evaluate the sequence, period, and carry-over effects. Crossover design is a type of longitudinal study, and the LME model has been widely used to analyze the data in longitudinal studies [33].

Patients were entered into the model as a random effect (nested in different sequences) to control within-subject effects. The sequence effect, period effect, carry-over effect (in the form of the interaction between time and group), and treatment effect with adjustment for the baseline values of outcome were examined.

This analysis was performed using SPSS software (version 24). The tests were considered two-tailed, and the significance level was set to 0.05. The LME analysis method is robust to the minor violation of the normality assumption. Therefore, this method can be used for the NRS score variable (i.e., an ordinal variable) [34–36]. Due to observing a significant carry-over effect, the analysis was performed only on the first-time (time 1) data. Based on the independence of the study groups, an analysis of covariance (ANCOVA) was used to compare the interventions’ effects [37].

## Results

According to the inclusion criteria, 41 patients under treatment with HD were included in the study. None of the patients was excluded and left the treatment half-finished. The mean and standard deviation of age and HD treatment duration were 52.98 ± 14.10 and 3.8 ± 3.1 years, respectively. In addition, 25 patients (61%) in this study were male. The results showed a significant interaction between time and group (P = 0.007). In other words, there were carry-over effects. The period (P = 0.67) and sequence (P = 0.36) effects were not significant (Table 1).

**Table 1:** The results of the linear mixed-effect model to assess group, carry over, period, and sequence effect on final pain with adjusted for baseline pain

Source	Numerator df	Denominator df	F	Sig.
Intercept	1	37.027	3.211	0.081
Group	2	76	5.123	0.008
Period	1	76.000	0.173	0.679
Sequence	2	56.648	1.028	0.364
Carry-over effect	2	76	5.315	**0.007**
Baseline pain	1	37.000	22.600	0.000

Dependent Variable: Pain

Cooling spray group (A) first (Time 1) received cooling spray; then after 2 weeks (Time 2), the group received lidocaine spray; finally, after 2 weeks (Time 3), the group received placebo spray. Lidocaine spray group (B) first (Time 1) received lidocaine spray; then, after 2 weeks (Time 2), the group received placebo spray; finally, after 2 weeks (Time 3), the group received cooling spray. Placebo spray group (C) first (Time 1) received placebo spray; then, after 2 weeks (Time 2), the group received cooling spray; finally, after 2 weeks (Time 3), the group received lidocaine spray.

**Fig. 2:**
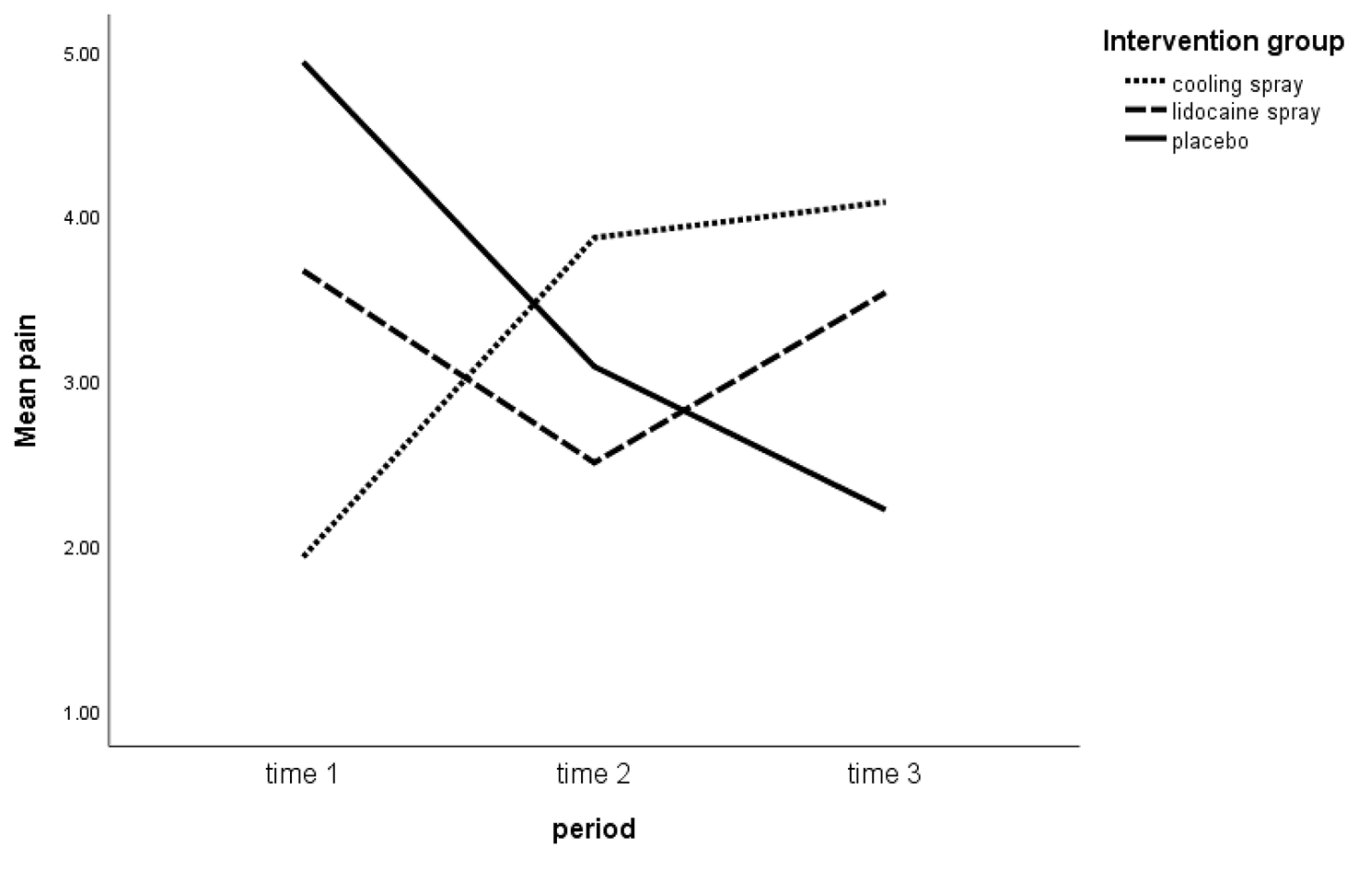
The pain score according to periods and groups of the study

As shown in Fig. 2, at the time, group A had the lowest pain score, which was after receiving the cooling spray. At the time 2, group B had the lowest pain score, which was after receiving the placebo spray; at time 3, group C had the lowest pain score, which was after receiving the cooling spray. Both intervention groups had significantly lower pain than the placebo group (P = 0.008), indicating a significant group effect. Cooling spray intervention significantly reduced pain, compared to placebo (P = 0.002); this reduction was not significant for the lidocaine spray group (P = 0.052). However, due to the existing carry-over effect, the effect obtained for the intervention was biased, and the net effect of the intervention could not be deduced from the analysis of all available data. Therefore, in this case, to avoid biased comparison between the groups, only observations of time 1 with adjustment for baseline values were used to evaluate the effect of the intervention [37]. The ANCOVA was the statistical method used for this analysis. The results of this comparison indicated that at the time 1, when there were no period and carry-over effects, by adjusting for baseline values, the type of intervention showed a significant effect on pain intensity (P-value for group = 0.01); accordingly, based on the results of multiple comparisons by the Bonferroni method, patients with cooling spray intervention reported 2.29 less pain score on average than patients with placebo intervention (B=-2.29, 95% CI: -4.17 to -0.43), which was statistically significant at the level of 0.05 (P = 0.012); however, it was not significant for lidocaine spray intervention, compared to placebo intervention (B=-0.68, 95% CI: -2.59 to 1.22) (P = 1). Additionally, patients receiving cooling spray reported a 1.61 lower pain score than those receiving lidocaine spray; nevertheless, this difference was not statistically significant (95% CI: -0.26 to 3.48; P = 0.113).

## Discussion

The present study was conducted to compare the effects of cooling and lidocaine sprays on needle insertion pain in HD patients using a crossover design. Because pain is subjective and pain is what the patient tells us according to the literature, it is difficult to compare patients’ pain scores to each other. On the other hand, patients undergoing HD frequently experience needle pain, which also affects their perception of pain; as a result, comparing the pain score of patients after receiving various interventions to themselves creates a more accurate picture of the effect of the intervention on pain control. Therefore, a crossover design was used in this study in a way that the participants can be compared to themselves, controlling for any possible confounding variables.

Moreover, this crossover design allows the comparison of various interventions. Although it was impossible to compare the pain scores at different times and after different interventions due to a carry-over effect, the present study results can help supplement the existing knowledge regarding cooling and lidocaine sprays. On the other hand, despite a two-week wash-out time, a carry-over effect in the crossover design was a significant issue that requires further investigation and more evidence on time needed to wash out the effects of similar interventions on pain in future studies.

The interventions’ effects were compared for the first time, controlling for baseline scores. Although the results showed no significant difference between cooling spray and lidocaine spray interventions, the difference in the scores of the two interventions was significant. More reliable results can be obtained by conducting further studies with a more extended wash-out period and a larger sample size.

The effects of cooling spray and lidocaine in various forms have been investigated in different studies, and different results have been reported. For example, Page and Taylor’s study on venous cannulation-induced pain showed that the pain reduction of cooling spray was less than topical lidocaine [31]. Additionally, the results of Celik et al.’s study showed that after using the Eutectic Mixture of Local Anesthetics (EMLA) cream and cooling spray, patients reported moderate to severe pain caused by HD needles equally and without significant differences [32], which is somewhat different from the results of the present study.

Moreover, Dalvandi et al.’s study revealed that the pain after receiving a cooling spray was significantly less than the control group (in the case of cannulation without any intervention) and more than the EMLA cream [29]. The aforementioned study showed that the cooling spray could not be as effective as the EMLA cream. This result is similar to the present study’s result regarding the effectiveness of a cooling spray in reducing pain, compared to a placebo.

The results of Moon et al.’s study investigating the effectiveness of cooling spray and lidocaine injection in propofol injection pain showed that both groups receiving cooling and lidocaine sprays experienced less pain than the control group; however, there was no significant difference between the two groups of lidocaine and cooling sprays in terms of pain score [38]. A study by Mace showed that a cooling spray was more successful in reducing venous needle insertion pain than a placebo [28]. These results are somewhat consistent with the results of the present study.

Limited studies have shown that cooling spray is ineffective in reducing pain [39, 40], which might be due to the different duration of cooling spray use, the distance of the spray from the skin, or the duration of needle insertion after using the spray. One of the issues raised regarding the use of a placebo is the placebo effect, which can cause an effect even in the absence of an intervention [20]. Although the pain score in the present study after using cooling and lidocaine sprays was lower than when using the placebo, it might be better to make this comparison in the absence of a placebo.

The NRS has inherent time and shortcomings. Furthermore, additional contributors to patients’ pain, such as catastrophizing tendencies, might be evident if additional or different rating scales were used to better clarify the patients’ phenotype.

Because HD patients need to be connected to the dialysis machine after needle insertion, it was difficult to evaluate the pain score immediately after the needle insertion; therefore, this evaluation was carried out in all patients within one minute after the needle insertion into the skin. The issue of whether the pain scores one minute after the needle insertion can be different from the score reported by the patient immediately after the needle insertion needs further investigation.

To the best of our knowledge, a limited number of studies compared the effect of cooling and lidocaine sprays in a crossover design. Therefore, compared to other studies, one of the advantages of this study is that it exposed a group of patients to three forms of intervention. On the other hand, the investigation of the sequence, period, and carry-over effects is one of the advantages of this study. The presence of a carry-over effect despite the 2-week wash-out period can raise one of the most important issues for further investigation in the field of pain studies.

This study has some limitations. Pain is subjective, and its evaluation can be associated with problems. On the other hand, patients undergoing HD treatment frequently experience needle pain that affects their perception of pain. Repeating a painful procedure (e.g., venous cannulation) might also shape patient expectations and preferences, and any intervention insulted by these issues might produce nocebo or placebo effects. The characteristics of vascular access can also be effective in pain perception. An attempt was made to cover this limitation using a crossover design. However, due to the significant carry-over effect, it was not possible to compare the scores after different interventions at different times in one group, which is another limitation of this study. Moreover, blinding was not possible in this study due to the cooling nature of the cooling spray and the apparent difference between lidocaine and placebo sprays. However, an attempt was made to ensure that covering lidocaine and placebo sprays was the same. Another limitation of this study was the small sample size.

## Conclusion

The results of this study indicated the effectiveness of cooling spray in reducing pain caused by HD needles. Due to its cost-effectiveness, immediate effect, and non-invasiveness, cooling spray can be an appropriate option for reducing pain in various techniques that are associated with pain. Since both interventions performed in this study were non-invasive techniques, these results can be helpful to be compared to other invasive pain reduction methods and make the right choice with fewer complications and more non-invasiveness. It is recommended to perform further studies to determine the wash-out period after non-invasive pain control interventions. In addition, it is required to carry out supplementary studies to compare the effects of lidocaine and cooling sprays.

## Data Availability

The datasets used and analyzed during the current study are not publicly available but are available from the corresponding author on reasonable request.

## Abbreviations

HD: Hemodialysis
AVF: Arteriovenous Fistula
NRS: Numerical Rating Scale
EMLA: Eutectic Mixture of Local Anesthetics
LME: Linear Mixed-Effect
ANCOVA: Analysis of Covariance
